# Crystal structure of dimethomorph

**DOI:** 10.1107/S2056989015014735

**Published:** 2015-08-12

**Authors:** Gihaeng Kang, Jineun Kim, Eunjin Kwon, Tae Ho Kim

**Affiliations:** a Department of Chemistry and Research Institute of Natural Sciences, Gyeongsang National University, Jinju 660-701, Republic of Korea

**Keywords:** crystal structure, dimethomorph, prop-2-en-1-one, fungicide

## Abstract

In the title compound, C_21_H_22_ClNO_4_ [systematic name: (*E*)-3-(4-chloro­phen­yl)-3-(3,4-di­meth­oxy­phen­yl)-1-(morpholin-4-yl)prop-2-en-1-one], which is the morpholine fungicide dimethomorph, the dihedral angles between the mean planes of the central chloro­phenyl and the terminal benzene and morpholine (r.m.s. deviation = 0.2233 Å) rings are 71.74 (6) and 63.65 (7)°, respectively. In the crystal, molecules are linked *via* C—H⋯O hydrogen bonds and weak Cl⋯π interactions [3.8539 (11) Å], forming a three-dimensional structure.

## Related literature   

For information on the fungicidal properties of the title compound, see: Xu *et al.* (2015 ▸). For related crystal structures, see: Chai & Liu (2011 ▸); Lu & Shi (2011 ▸).
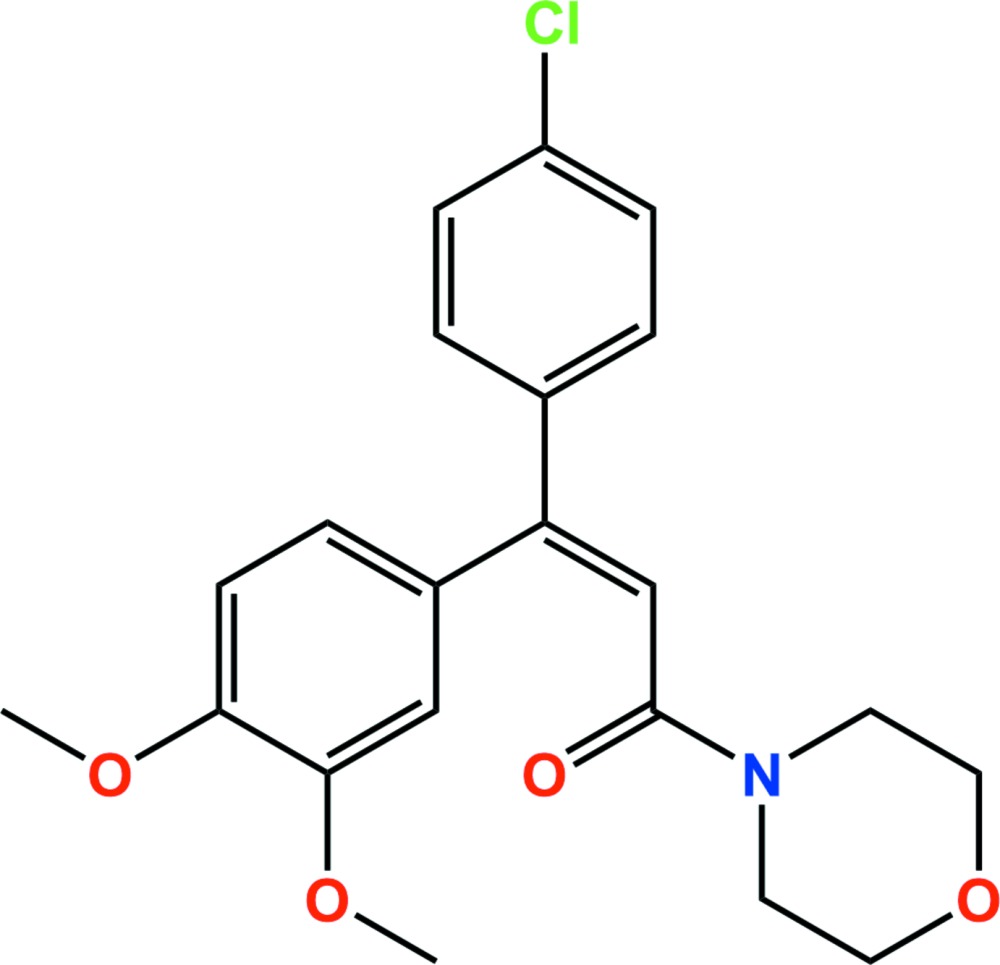


## Experimental   

### Crystal data  

C_21_H_22_ClNO_4_
*M* *_r_* = 387.84
Monoclinic, 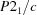

*a* = 6.6238 (2) Å
*b* = 13.2232 (4) Å
*c* = 21.4810 (7) Å
β = 97.1674 (19)°
*V* = 1866.77 (10) Å^3^
*Z* = 4
Mo *K*α radiation
μ = 0.23 mm^−1^
*T* = 173 K
0.38 × 0.06 × 0.03 mm

### Data collection  

Bruker APEXII CCD diffractometer
Absorption correction: multi-scan (*SADABS*; Bruker, 2013 ▸) *T*_min_ = 0.917, *T*_max_ = 0.993
18090 measured reflections
4276 independent reflections
3119 reflections with *I* > 2σ(*I*)
*R*_int_ = 0.047

### Refinement  

*R*[*F* ^2^ > 2σ(*F* ^2^)] = 0.048
*wR*(*F* ^2^) = 0.133
*S* = 1.04
4276 reflections
246 parameters
H-atom parameters constrained
Δρ_max_ = 0.27 e Å^−3^
Δρ_min_ = −0.53 e Å^−3^

### 

Data collection: *APEX2* (Bruker, 2013 ▸); cell refinement: *SAINT* (Bruker, 2013 ▸); data reduction: *SAINT*; program(s) used to solve structure: *SHELXS97* (Sheldrick, 2008 ▸); program(s) used to refine structure: *SHELXL2013* (Sheldrick, 2015 ▸); molecular graphics: *DIAMOND* (Brandenburg, 2010 ▸); software used to prepare material for publication: *SHELXTL* (Sheldrick, 2008 ▸).

## Supplementary Material

Crystal structure: contains datablock(s) global, I. DOI: 10.1107/S2056989015014735/hg5456sup1.cif

Structure factors: contains datablock(s) I. DOI: 10.1107/S2056989015014735/hg5456Isup2.hkl

**Fig. 1.**
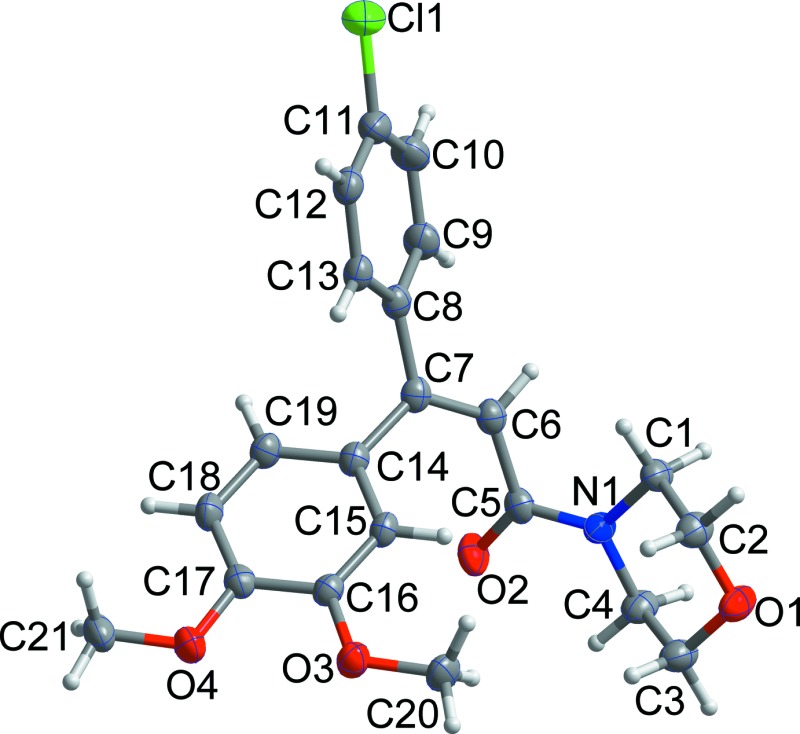
The asymmetric unit of the title compound with the atom numbering scheme. Displacement ellipsoids are drawn at the 50% probability level. H atoms are shown as small spheres of arbitrary radius.

**Fig. 2.**
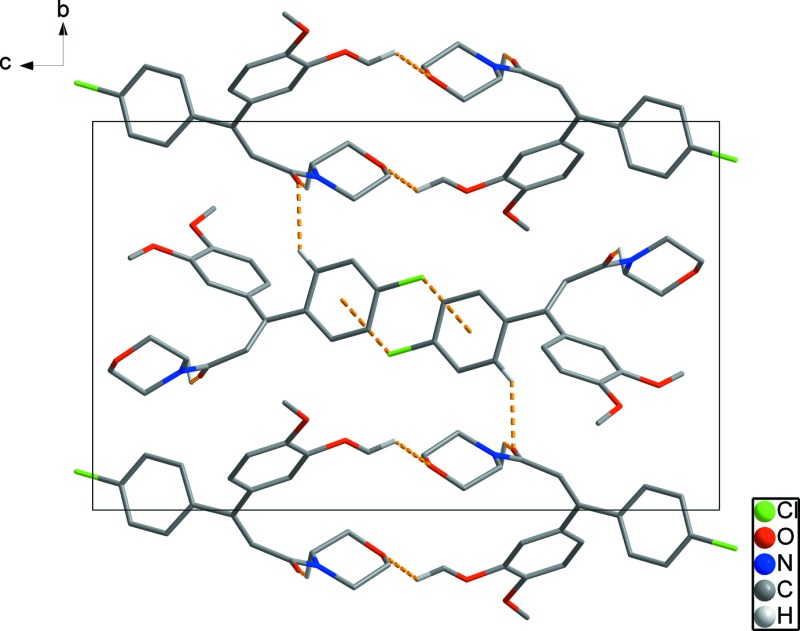
Crystal packing viewed along the a axis. The intermolecular interactions are shown as dashed lines.

. DOI: 10.1107/S2056989015014735/hg5456fig1.tif
The asymmetric unit of the title compound with the atom numbering scheme. Displacement ellipsoids are drawn at the 50% probability level. H atoms are shown as small spheres of arbitrary radius.

a . DOI: 10.1107/S2056989015014735/hg5456fig2.tif
Crystal packing viewed along the *a* axis. The inter­molecular inter­actions are shown as dashed lines.

CCDC reference: 1417163

Additional supporting information:  crystallographic information; 3D view; checkCIF report

## Figures and Tables

**Table 1:** Hydrogen-bond geometry (, )

*D*H*A*	*D*H	H*A*	*D* *A*	*D*H*A*
C1H1*B*O2^i^	0.99	2.53	3.167(2)	122
C13H13O2^ii^	0.95	2.38	3.166(2)	140
C20H20*B*O1^iii^	0.98	2.64	3.010(2)	103

Symmetry codes: (i) 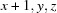; (ii) 
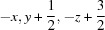
; (iii) 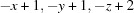.

## supplementary crystallographic information

### S1. Comment

Dimethomorph [systematic name: (*E*)-3-(4-chlorophenyl)-3-(3,4-dimethoxyphenyl)-1-(morpholin-4-yl)prop-2-en-1-one] is a morpholine fungicide that has been mainly applied on grapevines, apples, ginsengs, tomatoes, potatoes, cucumbers, Chinese cabbage and other crops. (Xu *et al.*, 2015). The dihedral angles between the planes of the central chlorophenyl and the terminal benzene and mean plane [r.m.s. deviation = 0.2233] of morpholine rings are 71.74 (6) and 63.65 (7)°, respectively. All bond lengths and bond angles are normal and comparable to those observed in similar crystal structures (Chai & Liu, 2011; Lu & Shi, 2011).

In the crystal structure (Fig. 2), C—H···O hydrogen bonds (Table 1) and weak intermolecular C11—Cl1···*Cg*1^iv^ (*Cg*1 is the centroid of the C8—C13 ring) interaction with a chlorophenyl ring are present, resulting in a three-dimensional network [for symmetry code: (iv), -*x*, -*y* + 1, -*z* + 1].

### S2. Experimental

The title compound was purchased from the Dr. Ehrenstorfer GmbH Company. Slow evaporation of a solution in CH_3_OH gave single crystals suitable for X-ray analysis.

### S3. Refinement

All H-atoms were positioned geometrically and refined using a riding model with d(C—H) = 0.98 Å, *U*_iso_ = 1.5*U*_eq_(C) for methyl group, d(C—H) = 0.99 Å, *U*_iso_ = 1.2*U*_eq_(C) for CH_2_ group, d(C—H) = 0.95 Å, *U*_iso_ = 1.2*U*_eq_(C) for C*sp*^2^—H and aromatic C—H.

### Crystal data

**Table:**

C_21_H_22_ClNO_4_	*F*(000) = 816
*M**_r_* = 387.84	*D*_x_ = 1.380 Mg m^−^^3^
Monoclinic, *P*2_1_/*c*	Mo *K*α radiation, λ = 0.71073 Å
*a* = 6.6238 (2) Å	Cell parameters from 3670 reflections
*b* = 13.2232 (4) Å	θ = 2.5–24.0°
*c* = 21.4810 (7) Å	µ = 0.23 mm^−^^1^
β = 97.1674 (19)°	*T* = 173 K
*V* = 1866.77 (10) Å^3^	Needle, colourless
*Z* = 4	0.38 × 0.06 × 0.03 mm

### Data collection

**Table:**

Bruker APEXII CCD diffractometer	3119 reflections with *I* > 2σ(*I*)
φ and ω scans	*R*_int_ = 0.047
Absorption correction: multi-scan (*SADABS*; Bruker, 2013)	θ_max_ = 27.5°, θ_min_ = 1.8°
*T*_min_ = 0.917, *T*_max_ = 0.993	*h* = −8→8
18090 measured reflections	*k* = −14→17
4276 independent reflections	*l* = −27→27

### Refinement

**Table:**

Refinement on *F*^2^	0 restraints
Least-squares matrix: full	Hydrogen site location: inferred from neighbouring sites
*R*[*F*^2^ > 2σ(*F*^2^)] = 0.048	H-atom parameters constrained
*wR*(*F*^2^) = 0.133	*w* = 1/[σ^2^(*F*_o_^2^) + (0.059*P*)^2^ + 0.6606*P*] where *P* = (*F*_o_^2^ + 2*F*_c_^2^)/3
*S* = 1.03	(Δ/σ)_max_ < 0.001
4276 reflections	Δρ_max_ = 0.27 e Å^−^^3^
246 parameters	Δρ_min_ = −0.53 e Å^−^^3^

### Special details

**Table:**

Geometry. All e.s.d.'s (except the e.s.d. in the dihedral angle between two l.s. planes) are estimated using the full covariance matrix. The cell e.s.d.'s are taken into account individually in the estimation of e.s.d.'s in distances, angles and torsion angles; correlations between e.s.d.'s in cell parameters are only used when they are defined by crystal symmetry. An approximate (isotropic) treatment of cell e.s.d.'s is used for estimating e.s.d.'s involving l.s. planes.

### Fractional atomic coordinates and isotropic or equivalent isotropic displacement parameters (Å^2^)

**Table:**

	*x*	*y*	*z*	*U*_iso_*/*U*_eq_	
Cl1	0.31366 (10)	0.59912 (5)	0.47448 (3)	0.0574 (2)	
O1	0.5956 (2)	0.38430 (11)	0.96279 (6)	0.0370 (4)	
O2	−0.0418 (2)	0.34526 (10)	0.82830 (7)	0.0370 (4)	
O3	−0.1413 (2)	0.67931 (10)	0.90317 (6)	0.0337 (3)	
O4	−0.4849 (2)	0.73290 (10)	0.84142 (6)	0.0368 (4)	
N1	0.2962 (2)	0.35718 (12)	0.85873 (7)	0.0281 (4)	
C1	0.5023 (3)	0.38939 (15)	0.84994 (9)	0.0305 (4)	
H1A	0.4969	0.4347	0.8131	0.037*	
H1B	0.5855	0.3296	0.8421	0.037*	
C2	0.5976 (3)	0.44402 (16)	0.90772 (9)	0.0347 (5)	
H2A	0.7399	0.4617	0.9027	0.042*	
H2B	0.5226	0.5078	0.9125	0.042*	
C3	0.3918 (3)	0.35949 (17)	0.97178 (9)	0.0364 (5)	
H3A	0.3150	0.4224	0.9772	0.044*	
H3B	0.3928	0.3187	1.0104	0.044*	
C4	0.2872 (3)	0.30099 (16)	0.91686 (9)	0.0335 (5)	
H4A	0.3542	0.2345	0.9142	0.040*	
H4B	0.1434	0.2890	0.9229	0.040*	
C5	0.1286 (3)	0.37008 (13)	0.81674 (9)	0.0267 (4)	
C6	0.1627 (3)	0.41177 (14)	0.75448 (9)	0.0277 (4)	
H6	0.2683	0.3819	0.7346	0.033*	
C7	0.0584 (3)	0.48755 (13)	0.72382 (9)	0.0263 (4)	
C8	0.1046 (3)	0.51370 (14)	0.65975 (9)	0.0274 (4)	
C9	0.1513 (3)	0.43952 (16)	0.61797 (9)	0.0354 (5)	
H9	0.1412	0.3702	0.6289	0.043*	
C10	0.2124 (3)	0.46527 (17)	0.56054 (10)	0.0411 (5)	
H10	0.2424	0.4140	0.5321	0.049*	
C11	0.2291 (3)	0.56604 (18)	0.54519 (10)	0.0378 (5)	
C12	0.1811 (3)	0.64122 (16)	0.58492 (10)	0.0350 (5)	
H12	0.1923	0.7103	0.5737	0.042*	
C13	0.1159 (3)	0.61476 (15)	0.64179 (9)	0.0303 (4)	
H13	0.0785	0.6664	0.6689	0.036*	
C14	−0.0896 (3)	0.55131 (13)	0.75275 (9)	0.0261 (4)	
C15	−0.0445 (3)	0.58425 (13)	0.81488 (8)	0.0254 (4)	
H15	0.0808	0.5648	0.8382	0.030*	
C16	−0.1772 (3)	0.64407 (13)	0.84292 (8)	0.0255 (4)	
C17	−0.3626 (3)	0.67311 (13)	0.80926 (9)	0.0278 (4)	
C18	−0.4093 (3)	0.64144 (14)	0.74781 (9)	0.0304 (4)	
H18	−0.5351	0.6606	0.7247	0.037*	
C19	−0.2734 (3)	0.58161 (14)	0.71962 (9)	0.0293 (4)	
H19	−0.3067	0.5612	0.6772	0.035*	
C20	0.0428 (3)	0.64921 (17)	0.93982 (9)	0.0369 (5)	
H20A	0.0476	0.5753	0.9428	0.055*	
H20B	0.0485	0.6782	0.9820	0.055*	
H20C	0.1591	0.6734	0.9199	0.055*	
C21	−0.6601 (3)	0.77604 (16)	0.80687 (10)	0.0388 (5)	
H21A	−0.6192	0.8187	0.7733	0.058*	
H21B	−0.7324	0.8172	0.8349	0.058*	
H21C	−0.7499	0.7220	0.7885	0.058*	

### Atomic displacement parameters (Å^2^)

**Table:**

	*U*^11^	*U*^22^	*U*^33^	*U*^12^	*U*^13^	*U*^23^
Cl1	0.0525 (4)	0.0813 (5)	0.0412 (3)	0.0133 (3)	0.0173 (3)	0.0147 (3)
O1	0.0326 (8)	0.0484 (8)	0.0281 (7)	−0.0095 (7)	−0.0041 (6)	0.0043 (6)
O2	0.0226 (7)	0.0352 (7)	0.0529 (9)	−0.0019 (6)	0.0031 (6)	0.0124 (7)
O3	0.0356 (8)	0.0358 (7)	0.0283 (7)	0.0058 (6)	−0.0015 (6)	−0.0037 (6)
O4	0.0333 (8)	0.0410 (8)	0.0362 (8)	0.0113 (6)	0.0048 (6)	0.0003 (6)
N1	0.0243 (8)	0.0340 (9)	0.0255 (8)	−0.0049 (7)	0.0017 (7)	0.0040 (7)
C1	0.0235 (10)	0.0395 (11)	0.0283 (10)	−0.0039 (8)	0.0028 (8)	0.0010 (9)
C2	0.0310 (11)	0.0399 (11)	0.0325 (11)	−0.0102 (9)	0.0011 (9)	0.0024 (9)
C3	0.0363 (12)	0.0462 (12)	0.0270 (10)	−0.0058 (10)	0.0045 (9)	0.0025 (9)
C4	0.0324 (11)	0.0373 (11)	0.0309 (11)	−0.0074 (9)	0.0045 (9)	0.0065 (9)
C5	0.0241 (9)	0.0211 (9)	0.0346 (10)	0.0003 (7)	0.0028 (8)	−0.0010 (8)
C6	0.0238 (9)	0.0271 (9)	0.0313 (10)	−0.0006 (8)	−0.0001 (8)	−0.0017 (8)
C7	0.0236 (9)	0.0252 (9)	0.0285 (10)	−0.0032 (8)	−0.0027 (8)	−0.0029 (8)
C8	0.0228 (9)	0.0302 (10)	0.0279 (10)	0.0004 (8)	−0.0020 (8)	−0.0016 (8)
C9	0.0378 (12)	0.0332 (10)	0.0347 (11)	0.0031 (9)	0.0020 (9)	−0.0045 (9)
C10	0.0394 (12)	0.0484 (13)	0.0361 (12)	0.0060 (10)	0.0077 (10)	−0.0077 (10)
C11	0.0301 (11)	0.0539 (13)	0.0298 (11)	0.0056 (10)	0.0046 (9)	0.0057 (10)
C12	0.0290 (11)	0.0387 (11)	0.0360 (11)	0.0038 (9)	−0.0013 (9)	0.0073 (9)
C13	0.0279 (10)	0.0313 (10)	0.0299 (10)	0.0036 (8)	−0.0031 (8)	0.0015 (8)
C14	0.0251 (9)	0.0232 (9)	0.0291 (10)	−0.0019 (7)	−0.0003 (8)	0.0024 (8)
C15	0.0236 (9)	0.0229 (9)	0.0278 (10)	−0.0012 (7)	−0.0032 (8)	0.0029 (7)
C16	0.0270 (10)	0.0235 (9)	0.0253 (10)	−0.0024 (7)	0.0008 (8)	0.0025 (7)
C17	0.0250 (10)	0.0245 (9)	0.0343 (11)	−0.0001 (8)	0.0051 (8)	0.0042 (8)
C18	0.0224 (9)	0.0326 (10)	0.0345 (11)	0.0010 (8)	−0.0033 (8)	0.0043 (8)
C19	0.0281 (10)	0.0304 (10)	0.0279 (10)	−0.0015 (8)	−0.0024 (8)	−0.0010 (8)
C20	0.0337 (11)	0.0473 (12)	0.0275 (11)	0.0019 (10)	−0.0055 (9)	−0.0033 (9)
C21	0.0278 (10)	0.0437 (12)	0.0458 (13)	0.0082 (9)	0.0084 (9)	0.0084 (10)

### Geometric parameters (Å, º)

**Table:**

Cl1—C11	1.739 (2)	C8—C9	1.390 (3)
O1—C2	1.424 (2)	C8—C13	1.395 (3)
O1—C3	1.426 (2)	C9—C10	1.388 (3)
O2—C5	1.230 (2)	C9—H9	0.9500
O3—C16	1.368 (2)	C10—C11	1.380 (3)
O3—C20	1.423 (2)	C10—H10	0.9500
O4—C17	1.378 (2)	C11—C12	1.373 (3)
O4—C21	1.417 (2)	C12—C13	1.390 (3)
N1—C5	1.351 (2)	C12—H12	0.9500
N1—C4	1.461 (2)	C13—H13	0.9500
N1—C1	1.464 (2)	C14—C19	1.390 (3)
C1—C2	1.505 (3)	C14—C15	1.400 (3)
C1—H1A	0.9900	C15—C16	1.376 (3)
C1—H1B	0.9900	C15—H15	0.9500
C2—H2A	0.9900	C16—C17	1.398 (3)
C2—H2B	0.9900	C17—C18	1.383 (3)
C3—C4	1.505 (3)	C18—C19	1.392 (3)
C3—H3A	0.9900	C18—H18	0.9500
C3—H3B	0.9900	C19—H19	0.9500
C4—H4A	0.9900	C20—H20A	0.9800
C4—H4B	0.9900	C20—H20B	0.9800
C5—C6	1.490 (3)	C20—H20C	0.9800
C6—C7	1.342 (3)	C21—H21A	0.9800
C6—H6	0.9500	C21—H21B	0.9800
C7—C14	1.487 (3)	C21—H21C	0.9800
C7—C8	1.487 (3)		
			
C2—O1—C3	110.32 (15)	C8—C9—H9	119.5
C16—O3—C20	117.63 (15)	C11—C10—C9	119.3 (2)
C17—O4—C21	117.58 (15)	C11—C10—H10	120.3
C5—N1—C4	121.14 (16)	C9—C10—H10	120.3
C5—N1—C1	125.36 (16)	C12—C11—C10	121.2 (2)
C4—N1—C1	113.35 (15)	C12—C11—Cl1	119.04 (17)
N1—C1—C2	109.58 (15)	C10—C11—Cl1	119.71 (17)
N1—C1—H1A	109.8	C11—C12—C13	119.03 (19)
C2—C1—H1A	109.8	C11—C12—H12	120.5
N1—C1—H1B	109.8	C13—C12—H12	120.5
C2—C1—H1B	109.8	C12—C13—C8	121.15 (19)
H1A—C1—H1B	108.2	C12—C13—H13	119.4
O1—C2—C1	111.87 (16)	C8—C13—H13	119.4
O1—C2—H2A	109.2	C19—C14—C15	117.86 (17)
C1—C2—H2A	109.2	C19—C14—C7	122.03 (17)
O1—C2—H2B	109.2	C15—C14—C7	120.10 (17)
C1—C2—H2B	109.2	C16—C15—C14	121.60 (17)
H2A—C2—H2B	107.9	C16—C15—H15	119.2
O1—C3—C4	111.29 (16)	C14—C15—H15	119.2
O1—C3—H3A	109.4	O3—C16—C15	124.38 (17)
C4—C3—H3A	109.4	O3—C16—C17	115.68 (17)
O1—C3—H3B	109.4	C15—C16—C17	119.94 (17)
C4—C3—H3B	109.4	O4—C17—C18	125.23 (17)
H3A—C3—H3B	108.0	O4—C17—C16	115.56 (17)
N1—C4—C3	110.16 (16)	C18—C17—C16	119.21 (18)
N1—C4—H4A	109.6	C17—C18—C19	120.49 (18)
C3—C4—H4A	109.6	C17—C18—H18	119.8
N1—C4—H4B	109.6	C19—C18—H18	119.8
C3—C4—H4B	109.6	C14—C19—C18	120.89 (18)
H4A—C4—H4B	108.1	C14—C19—H19	119.6
O2—C5—N1	121.99 (18)	C18—C19—H19	119.6
O2—C5—C6	121.71 (17)	O3—C20—H20A	109.5
N1—C5—C6	116.24 (16)	O3—C20—H20B	109.5
C7—C6—C5	126.17 (17)	H20A—C20—H20B	109.5
C7—C6—H6	116.9	O3—C20—H20C	109.5
C5—C6—H6	116.9	H20A—C20—H20C	109.5
C6—C7—C14	122.97 (18)	H20B—C20—H20C	109.5
C6—C7—C8	118.35 (17)	O4—C21—H21A	109.5
C14—C7—C8	118.52 (16)	O4—C21—H21B	109.5
C9—C8—C13	118.25 (18)	H21A—C21—H21B	109.5
C9—C8—C7	121.42 (17)	O4—C21—H21C	109.5
C13—C8—C7	120.18 (17)	H21A—C21—H21C	109.5
C10—C9—C8	120.9 (2)	H21B—C21—H21C	109.5
C10—C9—H9	119.5		
			
C5—N1—C1—C2	132.71 (19)	Cl1—C11—C12—C13	−179.22 (15)
C4—N1—C1—C2	−51.7 (2)	C11—C12—C13—C8	1.9 (3)
C3—O1—C2—C1	−60.0 (2)	C9—C8—C13—C12	−2.9 (3)
N1—C1—C2—O1	55.2 (2)	C7—C8—C13—C12	172.68 (17)
C2—O1—C3—C4	59.7 (2)	C6—C7—C14—C19	−139.6 (2)
C5—N1—C4—C3	−132.19 (19)	C8—C7—C14—C19	45.1 (2)
C1—N1—C4—C3	52.0 (2)	C6—C7—C14—C15	41.5 (3)
O1—C3—C4—N1	−55.3 (2)	C8—C7—C14—C15	−133.79 (18)
C4—N1—C5—O2	8.1 (3)	C19—C14—C15—C16	0.5 (3)
C1—N1—C5—O2	−176.63 (17)	C7—C14—C15—C16	179.42 (16)
C4—N1—C5—C6	−169.19 (17)	C20—O3—C16—C15	−2.2 (3)
C1—N1—C5—C6	6.1 (3)	C20—O3—C16—C17	178.19 (16)
O2—C5—C6—C7	50.0 (3)	C14—C15—C16—O3	−179.46 (17)
N1—C5—C6—C7	−132.7 (2)	C14—C15—C16—C17	0.2 (3)
C5—C6—C7—C14	9.2 (3)	C21—O4—C17—C18	−8.5 (3)
C5—C6—C7—C8	−175.53 (17)	C21—O4—C17—C16	171.23 (17)
C6—C7—C8—C9	37.0 (3)	O3—C16—C17—O4	−0.4 (2)
C14—C7—C8—C9	−147.52 (18)	C15—C16—C17—O4	179.97 (16)
C6—C7—C8—C13	−138.47 (19)	O3—C16—C17—C18	179.34 (16)
C14—C7—C8—C13	37.0 (2)	C15—C16—C17—C18	−0.3 (3)
C13—C8—C9—C10	1.6 (3)	O4—C17—C18—C19	179.47 (17)
C7—C8—C9—C10	−173.99 (18)	C16—C17—C18—C19	−0.2 (3)
C8—C9—C10—C11	0.8 (3)	C15—C14—C19—C18	−1.0 (3)
C9—C10—C11—C12	−1.8 (3)	C7—C14—C19—C18	−179.93 (17)
C9—C10—C11—Cl1	177.89 (16)	C17—C18—C19—C14	0.9 (3)
C10—C11—C12—C13	0.5 (3)		

### Hydrogen-bond geometry (Å, º)

**Table:**

*D*—H···*A*	*D*—H	H···*A*	*D*···*A*	*D*—H···*A*
C1—H1*B*···O2^i^	0.99	2.53	3.167 (2)	122
C13—H13···O2^ii^	0.95	2.38	3.166 (2)	140
C20—H20*B*···O1^iii^	0.98	2.64	3.010 (2)	103

Symmetry codes: (i) *x*+1, *y*, *z*; (ii) −*x*, *y*+1/2, −*z*+3/2; (iii) −*x*+1, −*y*+1, −*z*+2.
