# Supplementary figures and images for: Crystal structure of dimethomorph
Source: Acta Crystallogr E Crystallogr Commun. 2015 Aug 12;71(Pt 9):o654. doi: 10.1107/S2056989015014735 (PMC4555370; doi:10.1107/S2056989015014735)

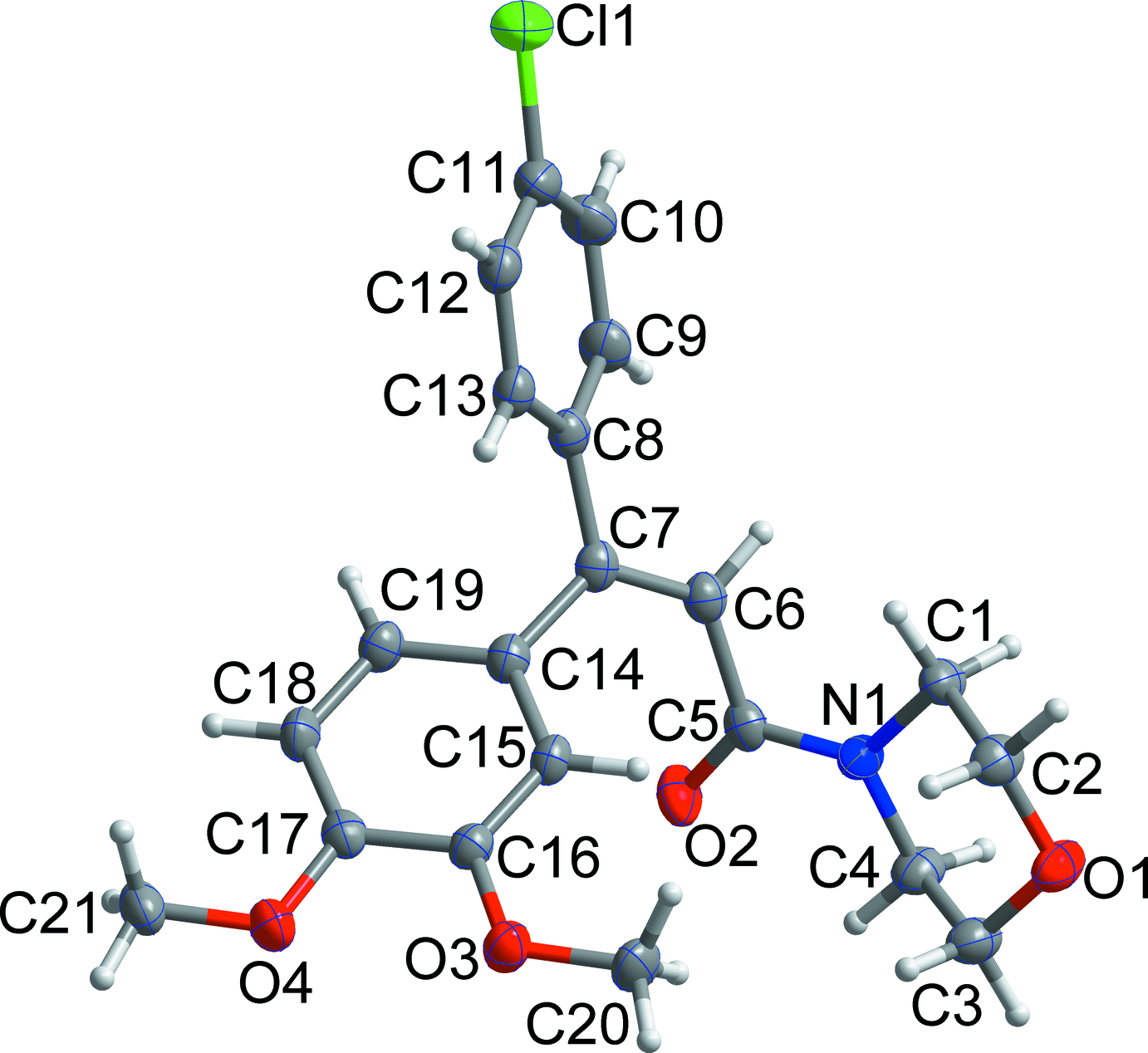

Supplement: Supplementary file 3 [file e-71-0o654-fig1.tif]

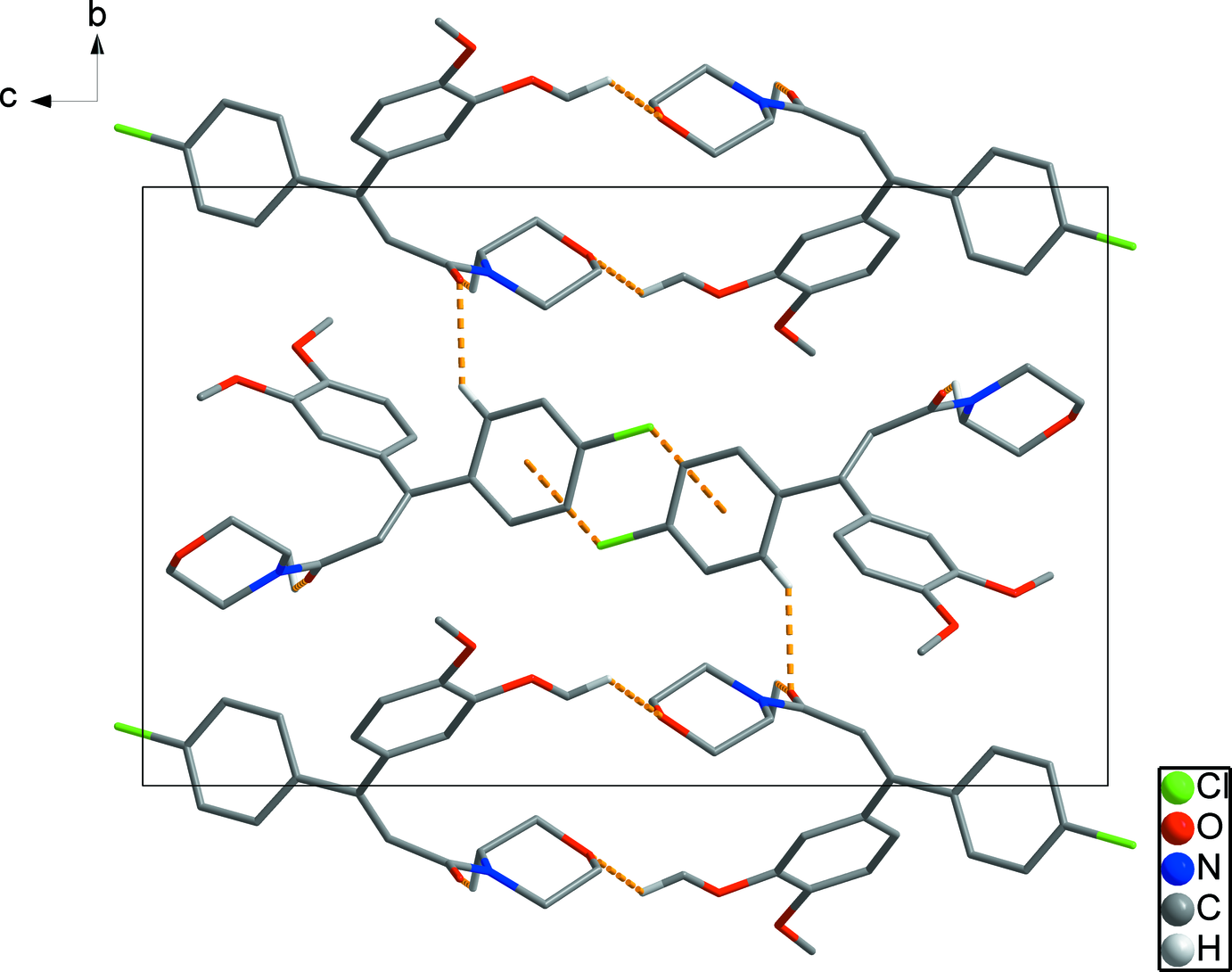

Supplement: Supplementary file 4 [file e-71-0o654-fig2.tif]
